# VIP and CRF reduce ADAMTS expression and function in osteoarthritis synovial fibroblasts

**DOI:** 10.1111/jcmm.12777

**Published:** 2016-01-28

**Authors:** Selene Pérez‐García, Mar Carrión, Irene Gutiérrez‐Cañas, Isidoro González‐Álvaro, Rosa P. Gomariz, Yasmina Juarranz

**Affiliations:** ^1^ Department of Cell Biology Faculty of Biology Complutense University Madrid Spain; ^2^ Reumatology Service Medical Research Institute La Princesa University Hospital Madrid Spain

**Keywords:** VIP, CRF, ADAMTS, osteoarthritis, synovial fibroblast, COMP, glycosaminoglycans

## Abstract

ADAMTS (a disintegrin and metalloproteinase with thrombospondin motifs) family is known to play an important role in the pathogenesis of osteoarthritis (OA), working on aggrecan degradation or altering the integrity of extracellular matrix (ECM). Thus, the main purpose of our study was to define the role of vasoactive intestinal peptide (VIP) and corticotrophin‐releasing factor (CRF), as immunoregulatory neuropeptides, on ADAMTS production in synovial fibroblasts (SF) from OA patients and healthy donors (HD). OA‐ and HD‐SF were stimulated with pro‐inflammatory mediators and treated with VIP or CRF. Both neuropeptides decreased ADAMTS‐4, ‐5, ‐7 and ‐12 expressions, aggrecanase activity, glycosaminoglycans (GAG), and cartilage oligomeric matrix protein (COMP) degradation after stimulation with fibronectin fragments (Fn‐fs) in OA‐SF. After stimulation with interleukin‐1β, VIP reduced ADAMTS‐4 and ‐5, and both neuropeptides decreased ADAMTS‐7 production and COMP degradation. Moreover, VIP and CRF reduced Runx2 and β‐catenin activation in OA‐SF. Our data suggest that the role of VIP and CRF on ADAMTS expression and cartilage degradation could be related to the OA pathology since scarce effects were produced in HD‐SF. In addition, their effects might be greater when a degradation loop has been established, given that they were higher after stimulation with Fn‐fs. Our results point to novel OA therapies based on the use of neuropeptides, since VIP and CRF are able to stop the first critical step, the loss of cartilage aggrecan and the ECM destabilization during joint degradation.

## Introduction

Control of the structure and function of the extracellular matrix (ECM) is a main role of ADAMTS (a disintegrin and metalloproteinase with thrombospondin motifs) family. They can be classified in four subgroups on the basis of their known substrates, for instance aggrecans, other proteoglycans, cartilage oligomeric matrix protein (COMP) or procollagen. Consequently, they have essential roles in tissue development and maintenance, and their dysregulation is associated with a number of diseases such as arthritis, cancer, atherosclerosis and central nervous system injury and disorders 1.

Osteoarthritis (OA) is a debilitating degenerative disease of articular joints, mainly characterized by articular cartilage degradation, subchondral bone alterations and localized inflammation 2. Osteoarthritis appears as a result of a progressive loss of aggrecan from cartilage, leading to exposure of the collagen matrix and its breakdown by metalloproteases 2, 3, 4. Within the subgroup of aggrecanases, ADAMTS‐4 and ‐5 play important roles in OA, emerging as therapeutic targets in arthritis 5. They are constitutively expressed in human chondrocytes and synovial fibroblasts (SF), and their expression is induced by pro‐inflammatory cytokines and products of matrix degradation as fibronectin fragments (Fn‐fs) 3, 6, 7. Other important molecule in the maintenance of cartilage is COMP that stabilizes ECM *via* specific interactions with matrix components such as collagen, aggrecan and fibronectin. Its degradation may also play a key role in the pathogenesis of arthritis 3, 8. ADAMTS‐7 and ‐12 belongs to the COMP‐cleaving enzymes subgroup. They are also produced by chondrocytes and SF and up‐regulated by pro‐inflammatory mediators [3, Pérez‐García et al., unpublished data]. Moreover, their overexpression is related to cartilage degenerative diseases and to disease progression in a mouse model of arthritis 9, 10.

Neuropeptides present in the joints are important mediators able to control several processes as inflammation, immunomodulation and cartilage or bone maintenance. Vasoactive intestinal peptide (VIP) and corticotropin‐releasing factor (CRF) are two neuropeptides present in the joint released from nerve ending, lymphocytes or SF, among other cells 11, 12. Vasoactive intestinal peptide exerts anti‐inflammatory and immunomodulatory actions in several autoimmune and inflammatory disorders 13, 14. Treatment with VIP significantly reduced incidence and severity of arthritis in a mouse experimental model, completely abrogating joint swelling and destruction of cartilage and bone 15, 16. In addition, VIP is able to reduce levels of some enzymes involved in ECM degradation in FLS from OA patients 17. On the other hand, CRF can exert both pro‐ and anti‐inflammatory functions depending on the type of receptors, tissues and disease phases 18, 19. Furthermore, some members of CRF family have shown a protective role on cartilage and bone cellular maintenance 20, 21.

All in all, ADAMTS play a key role in the cartilage destruction in OA, and their modulation is essential for the maintenance of the joints. Thus, the main purpose of our study was to define the role of VIP and CRF on ADAMTS produced by SF, as important cells also implicated in the maintenance of the cartilage ECM, in OA patients and healthy donors (HD). To our knowledge, this is the first study demonstrating that VIP and CRF can target the ADAMTS family *in vitro*.

## Patients and methods

### Patients and SF cultures

Synovial tissue was obtained from 20 active OA patients (16 women and 4 men) aged between 48 and 87 years, at the time of knee prosthetic replacement surgery. Patients had advanced disease and were diagnosed of primary OA, excluding trauma, inflammatory disease, and other structural causes of secondary OA. Control samples from HD were obtained from four patients (2 women and 2 men) aged between 35 and 72 years, diagnosed with meniscopathy, excluding inflammatory and rheumatic diseases, at the time of a knee arthroscopic evaluation. The study was performed according to the recommendations of the Declaration of Helsinki and was approved by the Clinical Research Ethics Committee of the Hospital La Princesa (Madrid, Spain). All biopsy samples were obtained after subjects gave their informed consent.

Synovial fibroblasts cultures were established by explant growth of synovial biopsies which were cultured in 10% heat‐inactivated foetal bovine serum (FBS)/DMEM (Lonza, Ibérica S.A.U., Barcelona, Spain) completed with 1% L‐glutamine and 1% antibiotic–antimycotic (Invitrogen, Carlsbad, CA, USA), at 37°C and 5% CO_2_. After three passages, residual contamination by macrophages was avoided and monocultures of SF were obtained and used for experiments until passage 8.

### Treatments of SF cultures

Healthy donors‐ and OA‐SF were cultured in serum‐free complete DMEM in the absence (untreated) or in the presence of the following agents: 10 ng/ml interleukin‐1β (IL‐1β; Immunotools GmbH, Friesoythe, Germany) or 10 nM Fn‐fs 45 kDa for 24 hrs. Fn‐fs were commercially predesigned from human plasma fibronectin (Sigma‐Aldrich, St Louis, MO, USA). These treatments were applied alone or in combination with 10 nM VIP (Polypeptide) or 10 nM CRF (Phoenix Pharmaceuticals, Inc, Karlsruhe, Germany).

### RNA extraction and RT‐qPCR for ADAMTS gene expression

Synovial fibroblasts were cultured in 100‐mm petri dishes (3 × 10^5^ cells/dish). Total RNA was obtained using Tri^®^Reagent (Sigma‐Aldrich). 2 μg RNA were reverse transcribed using a High Capacity cDNA Reverse Transcription Kit (Applied Biosystems, Foster City, CA, USA). Semiquantitative real‐time PCR analysis was performed with a TaqMan^®^ Gene Expression Master Mix with manufactured‐predesigned primers and probes for β‐actin (NM001101.3), ADAMTS‐4 (NM005099.4), ADAMTS‐5 (NM007038.3), ADAMTS‐7 (NM014272.3) and ADAMTS‐12 (NM030955.2) (Applied Biosystems). For relative quantification, we normalized the target gene expression to the housekeeping gene (β‐actin). Results were presented as the relative expression with respect to the untreated condition using the formula 2^−∆∆Ct^, as previously described 12.

### Quantification of ADAMTS in culture supernatant

Synovial fibroblasts were cultured in six‐well plates (6 × 10^4^ cells/well). The levels of ADAMTS were measured in the culture supernatants using a commercial ELISA kits for ADAMTS‐4 and ‐5 (Cloud‐Clone Corp, Houston, TX, USA), and ADAMTS‐7 and ‐12 (MyBioSource, San Diego, CA, USA).

### Aggrecanase activity assay

Aggrecanase activity was measured in the SF culture supernatants from 100‐mm petri dishes (3 × 10^5^ cells/dish) using a Sensitive Aggrecanase Activity ELISA kit (MD Bioproducts, Zürich, Switzerland), according to the manufacturer's instructions. Briefly, this assay consists in two modules. In the Aggrecanase Module, a modified interglobular domain (aggrecan‐IGD‐s) is digested with aggrecanases, and its proteolytic cleavage releases an aggrecan peptide (ARGSVIL‐peptide‐s), which is then quantified with antibodies in the ELISA Module.

### Glycosaminoglycans and COMP assays in cartilage‐SF co‐cultures

Release of glycosaminoglycans (GAGs) and COMP degradation products from cartilage tissue was measured in culture medium from wells containing SF cultured over cartilage explants. Osteoarthritis human cartilages were obtained from three patients undergoing total hip arthroplasty in Hospital del Mar (Barcelona, Spain). Fixed diameter (6 mm) and height (2 mm) sections were collected from cartilage areas without macroscopically signs of OA. The samples were frozen at −80°C and stored until testing. One explant per well was attached to a 24 well plate. Healthy donors‐ or OA‐SF were added drop‐wise on top of the cartilage surface (2 × 10^4^ SF/explant). After 3 hrs of incubation at 37°C, wells were filled with 1 ml DMEM 10% FBS, with the treatments described, and cultures were continued for 14 days. Culture supernatants were collected to detect the release of GAGs and COMP degradation products, using a Blyscan^™^ Sulfated Glycosaminoglycan Assay (Biocolor Ltd, County Antrim, Ireland, UK), and a Quantikine^®^ Human COMP Immunoassay (R&D Systems, Abingdon, OX, UK), respectively. Cartilage explants were placed in OCT and frozen in liquid nitrogen. Sections were prepared using a cryostat, placed on slides and stained with Alcian blue and Callejas's tricromic.

### Runx2 Transcription factor activity assay

Synovial fibroblasts were cultured in 150‐mm petri dishes (8 × 10^5^ cells/dish). A Nuclear Extract Kit (Active Motif, Rixensart, Belgium) was used for nuclear extract preparation, and the protein content was measured with a QuantiProTM BCA Assay Kit (Sigma‐Aldrich). The nuclear protein extracts (12 μg/well) were added to a 96‐well plate and Runx2 activity was measured using a TransAM^™^ AML‐3/Runx2 kit (Active Motif). We studied the time course of Runx2 activation after incubation with IL‐1β or Fn‐fs. Next, we performed the experiments at 60 min. or 30 min. respectively.

### β‐catenin assay

To detect β‐catenin levels, a β‐catenin (Total) and a (Phospho) InstantOneTM ELISA kits were used (eBioscience, San Diego, CA, USA) with SF cellular lysates. Briefly, SF cultured in 100‐mm petri dishes (3 × 10^5^ cells/dish) were scraped into PBS, centrifuged and resuspended in the Cell Lysis Buffer Mix. Protein content was measured using a QuantiProTM BCA Assay Kit (Sigma‐Aldrich). The levels of β‐catenin in the cellular lysates were measured after 60 min. of treatment, when the highest production of β‐catenin was observed.

### Statistical analysis

Samples were tested for normality by a Normality Test. Differences between means of different groups (one‐to‐one comparisons) were assessed using a Student's two‐tailed *t*‐test with *P* < 0.05 considered significant. Results are presented as the mean with a 95% confidence interval (CI) (lower limit, upper limit). Data were analysed with GraphPad Prism 6 (GraphPad Software, Inc., San Diego, CA, USA).

## Results

### VIP and CRF decrease ADAMTS‐4, ‐5, ‐7 and ‐12 expression and function after stimulation with Fn‐fs in SF from OA patients

**Figure 1:**
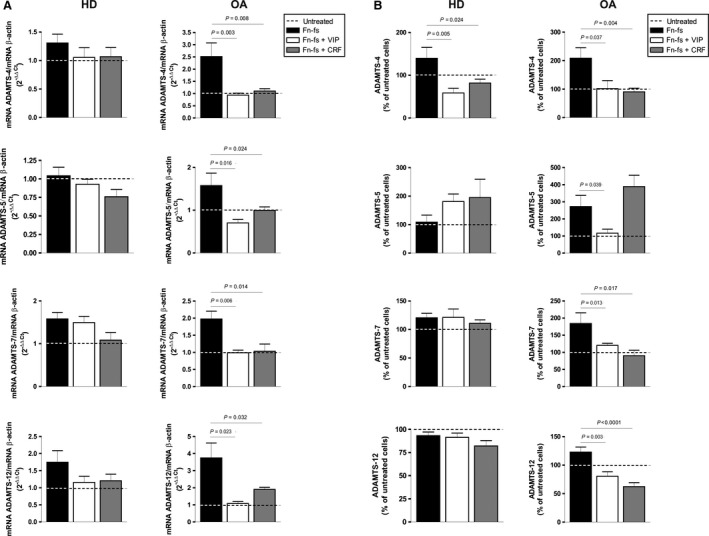
mRNA and protein expression of ADAMTS in Fn‐fs‐induced OA‐SF in the presence of VIP or CRF. (**A**) ADAMTS‐4, ‐5, ‐7 and ‐12 mRNA expression was measured after 24 hrs of treatment by RT‐qPCR, normalized to β‐actin and presented as the relative quantification with respect to the untreated cells using the formula 2^−∆∆Ct^ (see Patients and methods). Values are presented as the mean ± S.E.M. of HD (*n* = 4) (*left*) and OA (*n* = 7) (*right*), performed in triplicate. (**B**) The presence of ADAMTS‐4, ‐5, ‐7 and ‐12 in the supernatants was determined by ELISA after 24 hrs of treatment. Values are presented as the percentage of untreated cells (mean ± S.E.M.) of HD (*n* = 4) (*left*) and OA (*n* = 7) (*right*), performed in duplicate. Dashed lines represent the untreated condition. Significant differences between treatments are indicated by a bar with the *P*‐value above.

Fibronectin fragments are ECM degradation products that act as inflammatory mediators and contribute to joint destruction in OA 22. The presence of Fn‐fs in OA‐SF increased the expression of ADAMTS [Pérez‐García et al., unpublished data]. Our results showed that treatment with VIP and CRF reduced mRNA expression and protein secretion of ADAMTS‐4, ‐7 and ‐12 induced by Fn‐fs in OA‐SF. The mRNA expression and protein secretion of ADAMTS‐5 were also decreased by VIP, whereas CRF only decreased the mRNA expression of these aggrecanases (Fig. 1A and B, *right*). By contrast, treatment of HD‐SF with these neuropeptides after stimulation with Fn‐fs only reduced the ADAMTS‐4 protein production (Fig. 1A and B, *left*).

**Figure 2:**
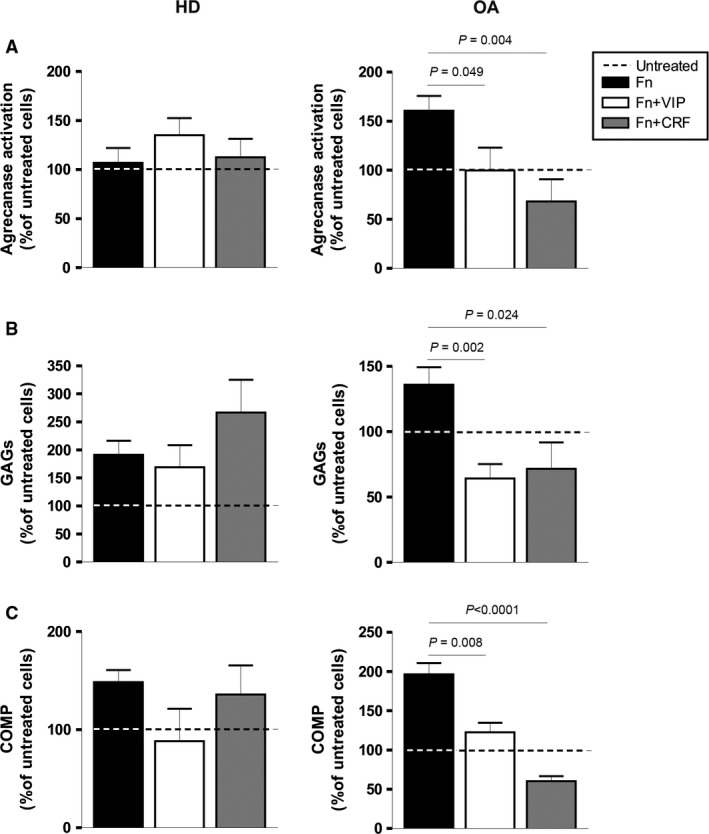
Aggrecanase activity and detection of GAGs and COMP degradation products in Fn‐fs‐induced OA‐SF in the presence of VIP or CRF. (**A**) Aggrecanase activity was measured in culture supernatants by an Aggrecanase Activity ELISA kit after 24 hrs of treatment. A mean of duplicate determinations was obtained for each HD (*n* = 4) (*left*) and OA (*n* = 5) (*right*). (**B**) GAGs in the supernatants from cartilage‐SF co‐cultures were detected after 14 days of treatment using a Blyscan^™^ Sulfated Glycosaminoglycan Assay. A mean of duplicate determinations was obtained for each HD (*n* = 3) (*left*) and OA (*n* = 3) (*right*). (**C**) COMP degradation products in the supernatants from cartilage‐SF co‐cultures were detected after 14 days of treatment using a Quantikine^®^ Human COMP Immunoassay. A mean of triplicate determinations was obtained for each HD (*n* = 3) (*left*) and OA (*n* = 3) (*right*). Dashed lines represent the untreated condition. Values are presented as the percentage of untreated cells (mean ± S.E.M.). Significant differences between treatments are indicated by a bar with the *P*‐value above.

To further dissect the role of VIP and CRF on ADAMTS family, we performed functional determinations of this metalloproteinases. Degradation of aggrecan is catalysed by ADAMTS‐4 and ‐5 2, 3, 4. Measurement of aggrecanase activity in culture supernatants showed a significant reduction in Fn‐fs‐induced OA‐SF after VIP and CRF treatments (Fig. 2A, *right*). The aggrecanase activity acts on aggrecan in the cartilage ECM releasing GAGs. We next studied the capacity of SF to degrade the cartilage by measuring GAG release in the supernatants of cartilage‐SF co‐cultures. When OA‐SF were treated with Fn‐fs, VIP and CRF significantly decreased the release of GAGs to the medium (Fig. 2B, *right*). None of the neuropeptides induced changes in the aggrecanase activity and in the GAGs release in HD‐SF. On the other hand, ADAMTS‐7 and ‐12 are associated with COMP, a non‐collagenous component of the cartilage ECM 3, 8. Cartilage oligomeric matrix protein degradation products from cartilage tissue were measured in supernatants from cartilage‐SF co‐cultures. Also, the presence of VIP and CRF in OA‐SF after induction with Fn‐fs significantly decreased the release of COMP fragments (Fig. 2C, *right*). No changes were observed in the case of HD‐SF (Fig. 2A–C, *left*).

### VIP and CRF differently modulate ADAMTS‐4, ‐5, ‐7 and ‐12 expression and function after stimulation with IL‐1β in SF from OA patients

**Figure 3:**
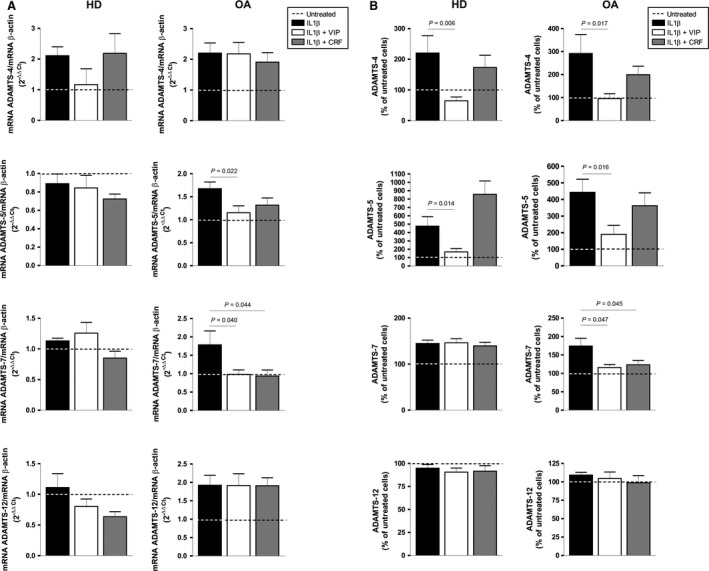
mRNA and protein expression of ADAMTS in IL‐1β‐induced OA‐SF in the presence of VIP or CRF. (**A**) ADAMTS‐4, ‐5, ‐7 and ‐12 mRNA expression after 24 hrs of treatment was measured by RT‐qPCR, normalized to β‐actin mRNA expression and presented as the relative quantification with respect to the untreated cells using the formula 2^−∆∆Ct^ (see Patients and methods). Values are presented as the mean ± S.E.M. of HD (*n* = 4) (*left*) and OA (*n* = 7) (*right*), performed in triplicate. (**B**) The presence of ADAMTS‐4, ‐5, ‐7 and ‐12 in the supernatants was determined by ELISA after 24 hrs of treatment. Values are presented as the percentage of untreated cells (mean ± S.E.M.) of HD (*n* = 4) (*left*) and OA (*n* = 7) (*right*), performed in duplicate. Dashed lines represent the untreated condition. Significant differences between treatments are indicated by a bar with the *P*‐value above.

To determine whether the effects of VIP or CRF on Fn‐fs‐induced ADAMTS could be extended to other pro‐inflammatory mediators present in the OA joints, we used the catabolic cytokine IL‐1β to induce ADAMTS expression in cultured SF. Previous studies have demonstrated that IL‐1β induces the expression of ADAMTS‐4, ‐5, ‐7 and ‐12 in OA‐SF [Pérez‐García et al., unpublished data, 23]. Here, we showed that VIP decreased the synthesis of ADAMTS‐4, ‐5 and ‐7. However, treatment with CRF showed a different pattern. Although CRF reduced both, mRNA expression and protein secretion of ADAMTS‐7, it did not show any effect on ADAMTS‐4 and ‐5 (Fig. 3A and B *right*). No changes were observed in ADAMTS‐12 after VIP or CRF treatments. The only effect observed in HD‐SF induced by IL‐1β was a reduction in the ADAMTS‐4 and ‐5 protein production after VIP treatment (Fig. 3A and B *left*).

**Figure 4:**
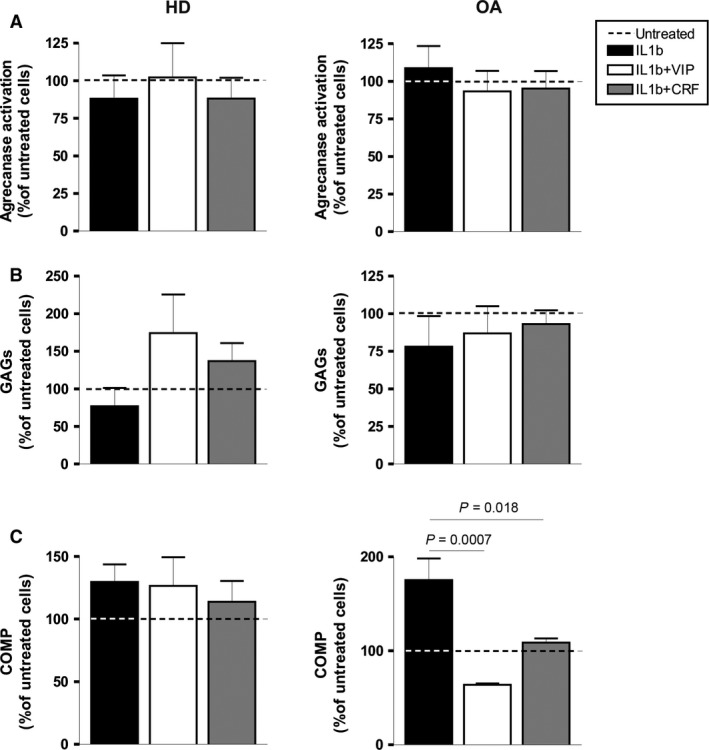
Aggrecanase activity and detection of GAGs and COMP degradation products in OA‐SF after stimulation with IL‐1β in the presence of VIP or CRF. (**A**) Aggrecanase activity was measured in culture supernatants by Aggrecanase Activity ELISA kit after 24 hrs of treatment. A mean of duplicate determinations was obtained for each HD (*n* = 4) (*left*) and OA (*n* = 5) (*right*). (**B**) GAGs in the supernatants from cartilage‐SF co‐cultures were detected after 14 days of treatment using a BlyscanTM Sulfated Glycosaminoglycan Assay. A mean of duplicate determinations was obtained for each HD (*n* = 3) (*left*) and OA (*n* = 3) (*right*). (**C**) COMP degradation products in the supernatants from cartilage‐SF co‐cultures were detected after 14 days of treatment using a Quantikine^®^ Human COMP Immunoassay. A mean of triplicate determinations was obtained for each HD (*n* = 3) (*left*) and OA (*n* = 3) (*right*). Dashed lines represent the untreated condition. Values are presented as the percentage of untreated cells (mean ± S.E.M.). Significant differences between treatments are indicated by a bar with the *P*‐value above.

Furthermore, the effect of VIP and CRF on ADAMTS‐4 and ‐5 expression after stimulation with IL‐1β was not confirmed at functional level. Aggrecanase activity and GAG release in the supernatants of OA cartilage‐SF co‐cultures showed similar values in the presence and absence of these neuropeptides (Fig. 4A and B, *right*). Conversely, the presence of VIP and CRF in IL‐1β‐induced OA‐SF significantly decreased the release of COMP fragments, related to ADAMTS‐7 and ‐12 actions (Fig. 4C, *right*). At functional level, no changes were observed in IL‐1β‐induced HD‐SF after VIP or CRF treatment (Fig. 4 A–C, *left*).

### VIP and CRF reduce activation of Runx2 and β‐catenin transcription factors in SF from OA patients

**Figure 5:**
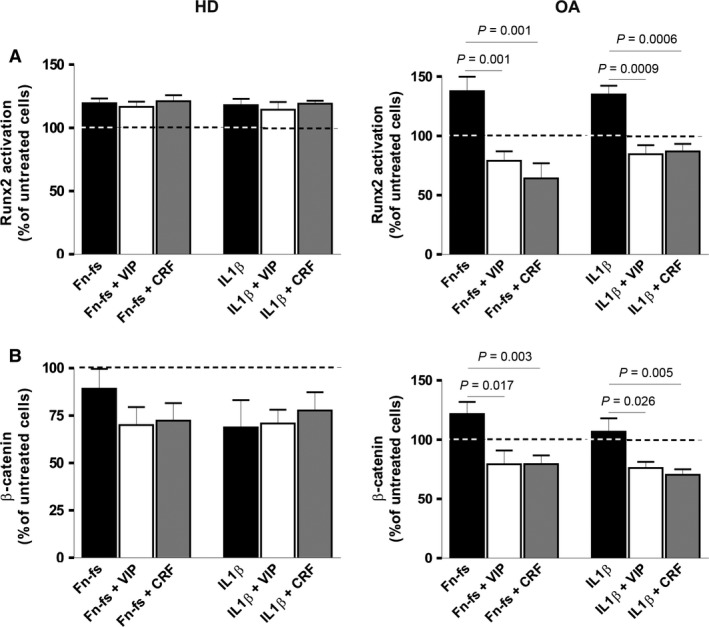
Runx2 and β‐catenin activation in OA‐SF after Fn‐fs or IL‐1β in the presence of VIP or CRF. (**A**) Runx2 activation was measured after treatment with IL‐1β for 60 min. or Fn‐fs for 30 min. in the presence or absence of VIP or CRF, in OA‐SF nuclear extracts by TransAM ^™^. A mean of duplicate determinations was obtained for each HD (*n* = 4) (*left*) and OA (*n* = 4) (*right*). (**B**) β‐catenin levels were detected after treatment with IL‐1β or Fn‐fs for 60 min. in the presence or absence of VIP or CRF, in OA‐SF cellular lysates by ELISA. A mean of duplicate determinations was obtained for each HD (*n* = 4) (*left*) and OA (*n* = 5) (*right*). Dashed lines represent the untreated condition. Values are presented as the percentage of untreated cells (mean ± S.E.M.). Significant differences between treatments are indicated by a bar with the *P*‐value above.

To elucidate the mechanism involved in the modulation of the ADAMTS family by VIP and CRF, we tested two transcription factors that regulate gene expression of some ADAMTS, Runx2 and β‐catenin 24, 25. Vasoactive intestinal peptide and CRF decreased the Runx2 activation induced by Fn‐fs and IL‐1β in OA‐SF (Fig. 5A, *right*). The other transcription factor studied, β‐catenin, showed the same profile (Fig. 5B, *right*). In this case, we measured the total β‐catenin that mainly represented the active form since the levels of phosphorylated β‐catenin were undetectable. No changes were observed in HD‐SF (Fig. 5A and B, *left*).

## Discussion

At present, this is the first study demonstrating the role of two endogenous neuropeptides present in the joint microenvironment, VIP and CRF, on ADAMTS family *in vitro*. Destruction of the ECM from the articular cartilage and subchondral bone in OA joints, is thought to be mediated by excessive proteolytic activity as a result of several events that take place in sequence 26. The ADAMTS family is known to play an important role in the pathogenesis of OA, working on aggrecan degradation or altering the integrity of the ECM 8, 27, 28, 29. The inhibition of these degradation enzymes can slow down or block disease progression. Thus, the characterization of mediators as neuropeptides, which can modulate these enzymes, is of great importance for both physiopathological and therapeutic perspectives.

Vasoactive intestinal peptide is a mediator shared by the neuroendocrine‐immune network, which is considered as a potential candidate for treatment in inflammatory and autoimmune diseases 13. Recently, VIP has been considered as a clinical biomarker in rheumatoid arthritis (RA) and OA, where low serum VIP levels were associated with worse prognosis 30, 31. In this way, VIP decreases pro‐inflammatory mediators and degradative proteases 11, 13, 17, 32. Moreover, protective effects of VIP upon bone and cartilage destruction have been described in experimentally induced arthritis 15, 16. Present results showed that VIP differentially affects ADAMTS‐4, ‐5, ‐7 and ‐12 in OA‐SF depending on the inflammatory mediator that induced their expression. When IL‐1β was present, the effect of VIP on ADAMTS‐4 and ‐5 protein production was not confirmed at functional level. The aggrecanase activity in SF and the GAG release in cartilage‐SF co‐cultures showed similar values in the presence or absence of VIP, probably because of the fact that IL‐1β did not induce the aggrecanase activity in SF [Pérez‐García et al., unpublished data, 33, 34]. However, VIP decreased expression and function of ADAMTS‐4 and ‐5 when they were induced by Fn‐fs, that was reflected in the reduction in the aggrecanase activity as well as in the decrease in GAGs liberated from the cartilage explants. In the case of ADAMTS associated with COMP degradation, VIP diminished ADAMTS‐7 mRNA expression and protein production in OA‐SF in the presence of both inflammatory mediators. Interestingly, the expression of ADAMTS‐7 is relatively late in OA development 8. However, ADAMTS‐12 transcript and protein were only decreased after Fn‐fs stimulation. Since VIP significantly decreased COMP degradation after both Fn‐fs and IL‐1β stimulation, altogether these data may suggest that the effects observed are mainly mediated by ADAMTS‐7. Fibronectin fragments result from progressive cartilage degeneration in injured joints and act as a continuous loop degrading cartilage through up‐regulation of cartilage degrading enzymes [Pérez‐García et al., unpublished data, 35]. Our data suggest that the role of VIP blocking ECM degradation through the modulation of ADAMTS expression could be related to the OA pathology since scarce effects were produced by VIP in HD‐SF. In addition, its actions might be higher when a degradation loop has been established given that the greater effects were observed after induction by Fn‐fs, present only in the late stages of OA.

Corticotrophin‐releasing factor has also an important role in the homoeostasis, both systematically and locally. An increasing body of data point to an anti‐inflammatory action of CRF *in vitro* 18, 36, 37. Besides, low CRF levels are clearly associated with disease severity in a human autoimmune disease 38. Another member of its family, urocortin, is implicated in osteoclast differentiation and is also related to the maintenance of the cartilage and bone in OA 20, 21. We observed that CRF had similar effects to VIP on ADAMTS‐7 and ‐12 expression and production after treatment with IL‐1β and Fn‐fs, and on ADAMTS‐4 and ‐5 expressions only after Fn‐fs stimulation, with no effect after induction with IL‐1β. At functional level, the effects observed with both neuropeptides were similar, decreasing the action of ADAMTS‐7 and ‐12 after both stimuli, and ADAMTS‐4 and‐5 functions only after Fn‐fs stimulation. These results may point out that CRF could block ECM degradation in OA modulating ADAMTS expression, in a similar way to VIP in late stages of the disease when a degradation loop has already been established. Moreover, a crosstalk between VIP and CRF in RA and OA has also been described 12. In addition, it is also important to consider that some pathophysiological conditions that take place during the disease can affect the release of this neuropeptides, modifying their levels 30, 31.

Runx 2 is an important transcription factor involved in both histogenesis and maintenance of the skeletal tissue, as well as in chondrocytes maturation and hypertrophy in OA 39, 40. On the other hand, β‐catenin is implicated in chondrocytes differentiation and skeletal maintenance, related to osteoblast differentiation and the blockade of osteoclastogenesis, which plays an important role in the pathology of OA 41, 42, 43. Several studies have demonstrated that Runx2 and β‐catenin are associated to ADAMTS‐4 and ‐5 24, 25, 44, nevertheless, there are no studies related to the transcription factors involved in ADAMTS‐7 and ‐12 gene expressions. Here, we showed that VIP and CRF reduced activation of Runx2 and β‐catenin transcription factors in OA‐SF after induction by IL‐1β and Fn‐fs in OA‐SF. As part of the anti‐inflammatory action of both neuropeptides 13, 14, 18, 19, the effects of VIP and CRF on ADAMTS could be mediated by the blockade of both transcription factors. Vasoactive intestinal peptide and CRF have demonstrated a protective role in cartilage and bone maintenance 15, 16, 20, 21, 45. This effect will depend on the cell type, the signalling pathways involved, and the interaction between the different cells present in the joint. Previous data reported the same effect of VIP on β‐catenin in renal cancer cells 46. In relation to CRF, it has only been described that Wnt/β‐catenin signalling pathway is increased by CRF in a mouse pituitary corticotroph cancer cells 47. Discrepancy with our results could be because of the different samples under study. Moreover, this is the first report demonstrating the modulation of Runx2 by VIP and CRF *in vitro*. Different studies have described that VIP activates PKA phosphorylating CREB inhibiting c‐Jun an AP‐1 in RA and OA‐SF 15, 48, 49. PKA/CREB signalling is also activated by CRF 50. As Runx2 is activated by c‐Jun 51, this signalling pathway could be implicated in the decrease in Runx2 observed after treatment with VIP and CRF. Moreover, it could impact in the effect observed on β‐catenin, as both signalling pathways are connected 52. On the other hand, it has been shown that the transcription factor NFκB plays an important role regulating different proinflammatory and destructive mediators in OA, including several matrix metalloproteases and ADAMTS 53, 54. In this sense, it has been reported that VIP decreases NFκB in synovial cells form CIA‐treated mice 15, 48. Then, the blockade of this signalling pathway could also be implicated in the decrease in the ADAMTS expression observed after treatment with VIP.

Regarding the effect of other neuropeptides on ADAMTS and consequently on ECM maintenance, data are scarce, even more in OA pathology. Only one study described that calcitonin decreases ADAMTS‐4 expression in a mouse model of OA 55, having a chondroprotective effect in a rabbit model of early OA 56. We have demonstrated that VIP and CRF are able to decrease ADAMTS expression and function in OA‐SF. Indeed, VIP reduces uPA (urokinase‐type plasminogen activator) in OA‐SF, a proteolytic enzyme upstream of the ADAMTS 17. The uPA‐ADAMTS axis is the main responsible for aggrecan cleavage and cartilage loss in OA. Matrix metalloproteinases (MMPs) participate downstream in this process and continue with the degradation of collagen 26. In this sense, VIP decreases MMP‐9 and ‐13 in OA‐SF after IL‐1β and Fn‐fs stimulation 17.

Although more studies are needed to elucidate the mechanism of action of VIP and CRF, our results point to novel targets to relieve the destruction of cartilage ECM in OA joints. Considering that VIP and CRF are able to modulate the first critical step, the loss of aggrecan in the destabilization of cartilage ECM, we corroborate the role of neuropeptides as potential therapeutic targets. Emerging therapies that directly or indirectly inhibit ADAMTS often lead to favourable outcome in OA patients.

## Conflicts of interest

A patent application (P201230827) for the use of VIP as a prognostic marker in autoimmune diseases has been deposited by the Biomedical Research Foundation of La Princesa University Hospital and Complutense University of Madrid at the Oficina Española de Patentes y Marcas. The full name of the patent application is: VIP use as a prognostic marker of autoimmune diseases.

## Author contribution

SPG performed the research; RPG and YJ designed the research study; SPG and MC analysed the data; IGA collected the samples; RPG, YJ, SPG and IGC wrote the paper.
